# The Epidemiology of Work-Related Injuries in Saudi Arabia Between 2016 and 2021

**DOI:** 10.7759/cureus.35849

**Published:** 2023-03-06

**Authors:** Saad M Asiri, Shady Kamel, Abdullah M Assiri, Abdulaziz S Almeshal

**Affiliations:** 1 Field Epidemiology Training Program, Ministry of Health, Riyadh, SAU; 2 Deputyship of Public Health, Ministry of Health, Riyadh, SAU

**Keywords:** construction injury, falls, saudi arabia, work-related injury, epidemiology

## Abstract

Background

Workplace safety has increased in many developed nations, but work-related injuries (WRIs) are on the rise. Globally, approximately 2.9 million fatal work-related injuries and diseases occurred in 2021, up from 1.1 million in 1999.

Method

This work employs a cross-sectional study using the open data published by the General Organization for Social Insurance (GOSI) on an annual basis for the years 2016 to 2021.

The data from one year in each category were compared with a reference year by using the index value method and simple linear regression. Additionally, the researchers calculated the injury-to-worker ratio for each year.

Aim

The aim of this study is to assess the trending epidemiology of work-related injuries in Saudi Arabia from 2016 to 2021 among insured workers and compare this trend to previous studies.

Result

A total of 1,009 work-related deaths wererecorded over the study period, averaging 168 per year. In comparison with non-Saudi workers, the Saudi workers' shares of injuries rose from 5.3% in 2016 to 10% in 2021. Injuries in the construction sector comprised 42%-48% of all injuries, followed by the commerce sector. In 2021, the highest percentage of injuries involved service occupations (47.5%), followed by the engineering sector. Between 2016 and 2018, the most common injury cause was falls (28.5% in 2018), and from 2020 to 2021, exposure to inanimate mechanical forces caused the most injuries (46%).

Conclusion

The study revealed that the trend of work-related injuries had decreased during the study period, consistent with the results of similar previous studies.

## Introduction

The International Labour Organization (ILO) defines a work-related injury (WRI) as "any personal injury, disease, or death resulting from a work-related accident" [1]. This type of injury differs from a work-related disease since the latter is a condition brought on by long-term exposure to risk factors associated with work activity. On the other hand, a work-related injury is "any abnormal condition or disorder resulting from an instantaneous event or exposure in the work environment," according to the work-related safety and health administration of the United States (OSHA) [2].

Since 1999, the ILO has produced global estimates of work-related injuries and diseases. All such estimates have indicated a significant increase in work-related fatal and nonfatal injuries throughout the last 20 years. Although workplace safety has increased in many developed nations, work-related injuries are on the rise everywhere. Globally, there were approximately 2.9 million fatal work-related injuries and diseases in 2021, up from 1.1 million in 1999, 2.3 million in 2014, and 2.8 million in 2017. Additionally, in 2021, an estimation of 956,000 deaths was caused by cardiovascular diseases, 799,000 by work-related cancer, and 493,000 by respiratory diseases. Overall, 312,000 people died because of work-related injuries [3].

The ILO estimates that 7,900 people die every day from diseases or injuries related to their jobs globally, with 11% of those deaths caused by WRI [3]. In the United States, six out of every 1,000 workers will suffer a fatal workplace injury during the course of a 40-year career [4]. The Global Estimates of Occupational Accidents And Work-Related Illnesses 2017 indicated that 92% of WRIs occurred in low- and middle-income countries despite underreporting [5].

Furthermore, WRI leads to not only fatalities and disabilities but also lost workdays and skilled personnel, lowering output. ILO estimates that workplace injuries and illnesses cost a staggering 5.4% of global gross domestic product (GDP) yearly or five trillion US dollars in direct and indirect costs, including lost workdays and compensation for injured workers, production delays, and medical expenses [1,6]. Numerous studies have been conducted on the financial impact of work-related illnesses and accidents. In 2020, the American National Safety Council (NSC) estimated that the cost of work-related illnesses and injuries in the United States was $163.9 billion [7]. Safe Work Australia reports that between 2012 and 2013, Australia spent A$61.8 billion (4.1% of GDP) on worker injuries and illnesses [8].

The burden of WRIs in Saudi Arabia's neighbors, including the Gulf Cooperation Council (GCC) nations, cannot be addressed without considering that WRIs are underreported in these nations, as in all developing nations. According to the Global Estimates of the Burden of Injury and Illness at Work in 2012 (Takala et al.), the only group where the level of injury reporting is close to expected levels is the group of developed countries. In contrast, reporting levels are generally low in other countries [9].

The incidence of fatalities in Jordan was determined to be 25.5 per 100,000 persons [10], and the number of injuries among oil field workers in Oman was determined to be 1,980 per 100,000 [11]. WRIs were reported to have a mortality rate of 136 per 100,000 workers per year in the United Arab Emirates in 2009. In that year, unintentional injuries were the second leading cause of death for foreign residents [12,13]. When compared with native workers in the GCC, migrant workers experience disproportionately higher rates of morbidity and mortality from WRIs and other illnesses [14]. A study from 2007 to 2008 in Qatar found that there were 86.7 annual injuries per 100,000 workers due to falls, with a fatality rate of 8.44%. An estimated $4.4 million was spent annually on these patients or an average of $15,735 [15].

A study conducted in Saudi Arabia from 2004 to 2016 utilizing the same data source as this study found that there were 6,562 work-related fatalities over the study period and an annual average of 505. In addition, the survey found that while WRIs had grown by about 300% by 2016, they were significantly more common among foreign workers than among Saudi nationals (93.5% versus 6.5%, respectively). Also, they found that construction had the greatest rate of WRIs at 46.5%, followed by retail at 23.8% and industry at 17.9%. That supports Takala et al.'s finding that there was a relative decline in WRIs per worker between 2004 and 2016 [9,16].

In Saudi Arabia, construction accidents and injuries are far more common than those in any other sector. For instance, over 51% of 69,241 work-related accidents and injuries reported in the private sector in 2014 were related to the construction business [17].

## Materials and methods

This is a cross-sectional study of all Saudi Arabian legalized workers' injuries using publicly available, year-by-year statistical information from the General Organization for Social Insurance (GOSI) website between 2016 and 2021 (http://www.gosi.gov.sa/).

At the end of the first quarter of 2022, GOSI had registered over nine million insured workers. This study included all workers who reported work-related injuries to GOSI between January 1, 2016, and December 31, 2021. This study's objectives necessitate the inclusion of all cases, and the data are well-defined and accessible; no sampling is required.

The variables that were used to organize data into Microsoft Excel (Microsoft® Corp., Redmond, WA) datasheets included total establishments, workers, work-related injuries and deaths, and the distribution of injuries according to demography, economic sectors, occupations, causes, and injury status.

However, since this study utilizes secondary summarized data from GOSI, the team encountered a problem when changing the classification of two data variables (causes and economic sector) during the periods of 2016-2018 and 2019-2021. For this reason, two separate analyses were conducted for the cause of injury variable, excluding the 2019 data due to poor quality. Additionally, the researchers reorganized the economic sector's data into a uniform classification using the National Classification of Economic Activities (ISIC4), previously used by GOSI [18].

In order to determine the relative importance of each variable, the team used an index value-calculating technique with 2016 as the reference year. These groups included construction industries in establishment economic activity, social service occupations in major work-related groups, falls as causes of work-related injuries, those cured without disability in recovery situations, the 30-34 age group, male workers, and non-Saudi workers. In Saudi Arabia [16], Pakistan [19], Turkey [20], and Korea [21], the index value-calculating approach was utilized to examine the epidemiology of work-related injury trends. The index value-calculating approach was employed in this study to investigate two different types of outcomes. The first was the distribution percentage of injuries by the status of work-related injuries, based on the reference year 2016. The second was the distribution percentage of work-related injuries based on each variable's reference group.

The following equation was used to determine the index for the year Y in relation to the reference year: (NY/Nref) × 100, where NY is the total number of work-related injuries during year Y and Nref is the number of injuries during the year used as a reference. Indices above 100 denote growth. For instance, Index 125 denotes a 25% rise from the reference year in the overall number of injuries, whereas Index 50 denotes a 50% drop. Additionally, the researchers calculated the injury-to-worker ratio, the total number of all work-related injuries (or specific ones) divided by the number of insured workers in a given year. Next, a simple linear regression analysis was used to generate a slope showing the downward or upward trend of work-related injuries from 2016 to 2021. The slope value (S) was used to describe the data's direction and steepness. A negative S value indicated a decrease in work-related injuries, whereas a positive (S) value indicated an increase.

According to GOSI, "the injury is considered an occupational injury in any of the following cases: accident sustained by the contributor during work or from which it resulted, accident sustained by the contributor on his way from his residence to his place of work and vice versa or while en route from his place of work to the place where he eats his meals or performs his prayers and vice versa, accident sustained by the contributor during his movements in order to perform the tasks entrusted to him by the employer, any disease sustained by the contributor found to be caused by work, any disease sustained by the contributor specified in the table of occupational diseases" [22].

The data from GOSI were reorganized in Excel sheet form according to the variable mentioned above, and then, the team used the Statistical Package for Social Sciences (SPSS) program (IBM SPSS Statistics, Armonk, NY) for data analysis.

The aim of this study is to evaluate the epidemiology of work-related injuries in Saudi Arabia from 2016 to 2021 among workers with insurance and to compare these results to those of prior studies.

## Results

Demographic distribution of work-related injuries

**Figure 1:**
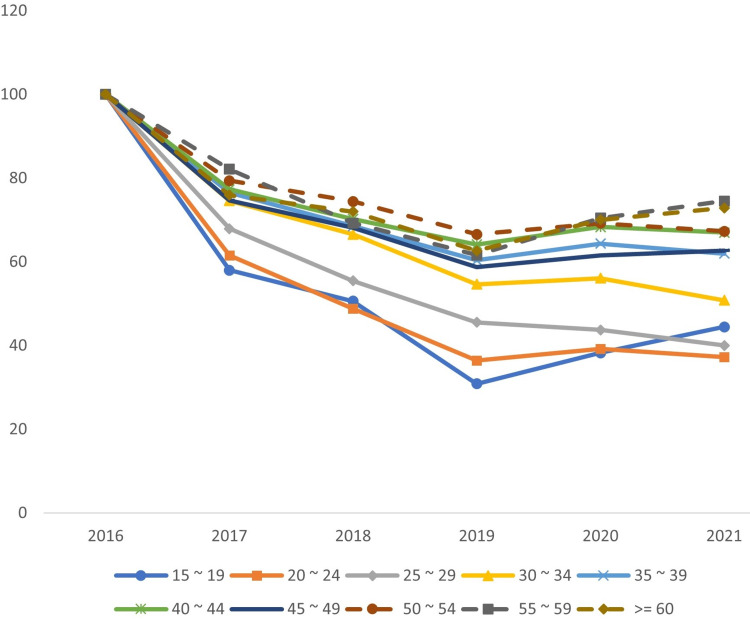
Trends in work-related injuries by age group in Saudi Arabia between 2016 and 2021

The age group with the highest percentage of injuries in 2021 was 30-34 years old. The indices of the age group below 30 decrease more significantly than those above 30 (Figure 1).

Comparing the different age groups to the 30-34 age group revealed a minimal decrease in slope (S) with S = -3.8 in 2016 and S = 1.7 in 2021 (Table 1).

**Table 1:** Index values of work-related injuries in Saudi Arabia by age group between 2016 and 2021 Index value by time, (30-34) = 100

Age groups	2016	2017	2018	2019	2020	2021
15-19	0.74	0.58	0.56	0.42	0.51	0.65
20-24	33.62	27.74	24.65	22.38	23.51	24.63
25-29	103.47	94.18	86.24	86.25	80.62	81.43
30-34	100	100	100	100	100	100
35-39	77.13	79.12	79.41	85.21	88.47	94.07
40-44	54.91	56.92	57.95	64.45	66.89	72.36
45-49	41.24	41.30	42.21	44.32	45.24	50.87
50-54	26.26	27.95	29.37	32.01	32.39	34.79
55-59	15.47	17.03	16.10	17.45	19.43	22.70
≥60	10.70	10.89	11.57	12.27	13.36	15.34
Slope	-3.77	-3.10	-2.67	-2.34	-2.06	-1.72

**Figure 2:**
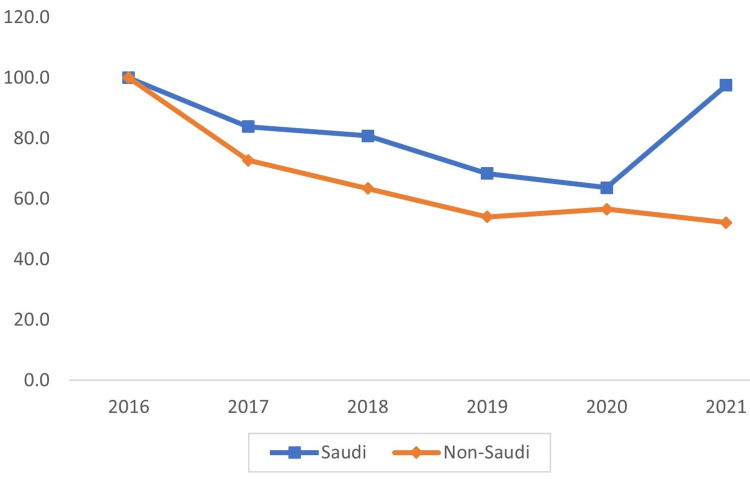
Trends in work-related injuries by nationality in Saudi Arabia between 2016 and 2021

Among the nationalities of injured workers, non-Saudi workers incurred the most injuries. However, the percentage of injuries decreased in the 2021 data compared to the 2016 data. Additionally, the injury-to-worker ratio decreased in both groups. Also, compared with non-Saudi workers (reference group), Saudi workers' percentage of injuries rose from 5.3% in 2016 to 10% in 2021 (Figure 2).

**Figure 3:**
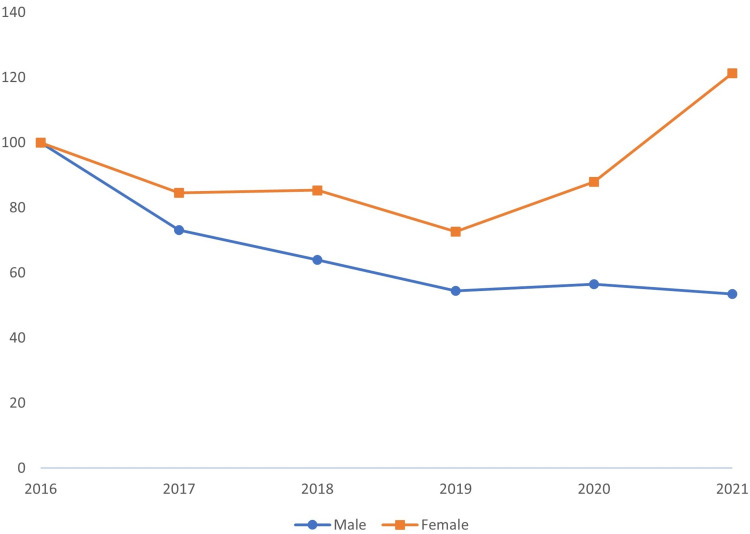
Trends in work-related injuries by gender in Saudi Arabia between 2016 and 2021

In terms of gender, the injury rate was greater for males than for females, and the ratio of injuries per worker was higher for males. However, there was a significant increase in female injuries in 2021 from 2016 (index value = 121.3). Additionally, there was a decrease in the injuries of male workers in the study period (slope = -8.3). The injury-to-worker ratio decreased in both groups over the study period (Table 2 and Figure 3).

**Table 2:** Distribution of injury-to-worker ratio by gender in Saudi Arabia between 2016 and 2021

Gender	2016	2017	2018	2019	2020	2021
Male	0.0052	0.0039	0.0038	0.0036	0.0037	0.0037
Female	0.0011	0.0009	0.0008	0.0007	0.0008	0.0009

Establishments, workers, injuries, and deaths

In the Kingdom of Saudi Arabia (KSA) between 2016 and 2021, Table 3 shows the number of establishments, insured workers, Saudi and foreign workers, work-related injuries, and work-related deaths. Over the course of the study, 1,009 deaths at work were reported or 168 each year on average. In the study period, the total number of establishments almost doubles from 2016 to 2021; a similar increase was observed among Saudi workers. Despite the 29% decrease in the number of foreign workers in 2021, the decrease in injuries for the same group was 50% in 2021, and injuries of Saudi workers decreased by only 2.5%, despite the increase of Saudi workers of more than 20%. The total injuries and deaths decreased by 46% and 84%, respectively, but the lowest numbers of total deaths occurred in 2020 (a 96% decrease: Table 3).

**Table 3:** Distribution of establishments, workers, work-related injuries, and deaths in Saudi Arabia between 2016 and 2021

Year	Establishments	Insured workers	Saudi workers	Non-Saudi workers	Work-related injuries	Injuries (Saudis)	Injuries (non-Saudi)	Work-related deaths
2016	455,641	10,290,903	1,708,714	8,582,189	50,602	2,573	48,029	506
2017	452,668	9,984,930	1,670,823	8,314,107	37,109	2,158	34,951	189
2018	483,846	9,129,006	1,733,560	7,395,446	32,557	2,080	30,477	115
2019	554,906	8,249,061	1,670,900	6,578,161	27,719	1,760	25,959	93
2020	629,790	8,324,039	1,678,093	6,645,946	28,813	1,639	27,174	22
2021	668,644	7,969,557	2,065,541	6,127,434	27,560	2,509	25,051	84

Distribution of work-related injuries by economic sector

**Figure 4:**
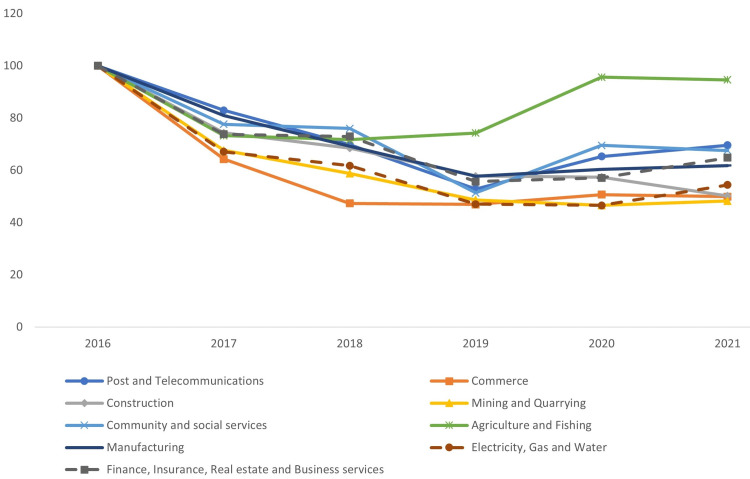
Trends in work-related injuries by economic sector in Saudi Arabia between 2016 and 2021

Injury rates in all economic sectors decreased by 31%-52% between 2016 and 2021, with the exception of agriculture and fishing, which decreased by only 5% in 2021. During the study period, the construction sector was responsible for 42%-48% of all injuries, followed by the commerce sector. The share of injuries in all sectors was almost the same each year, except for a slight increase in the agriculture and fishing shares (Figure 4).

The injury-to-worker ratio decreased in all sectors, but the decrease was more significant in the community and social service sector (45%) and much less significant in the agriculture and fishing sectors (6%). When comparing different sectors with the reference group (construction), the slope remained steady over the study period (Table 4).

**Table 4:** Index values of work-related injuries in Saudi Arabia by economic sector between 2016 and 2021 Index value by time, construction = 100

Economic sectors	2016	2017	2018	2019	2020	2021
Post and telecommunications	6.05	6.75	6.17	5.52	6.88	8.38
Commerce	47.49	41.08	32.85	38.50	41.96	47.20
Construction	100	100	100	100	100	100
Mining and quarrying	4.85	4.42	4.17	4.08	3.94	4.66
Community and social services	6.97	7.28	7.73	6.18	8.44	9.38
Agriculture and fishing	1.70	1.67	1.78	2.18	2.83	3.20
Manufacturing	37.70	41.04	38.10	37.64	39.61	46.44
Electricity, gas, and water	2.45	2.21	2.21	1.99	1.99	2.65
Finance, insurance, real estate, and business services	14.49	14.38	15.44	13.94	14.41	18.70
Slope	-3.82	-3.45	-3.02	-3.37	-3.53	-3.35

Distribution of work-related injuries by occupation

**Figure 5:**
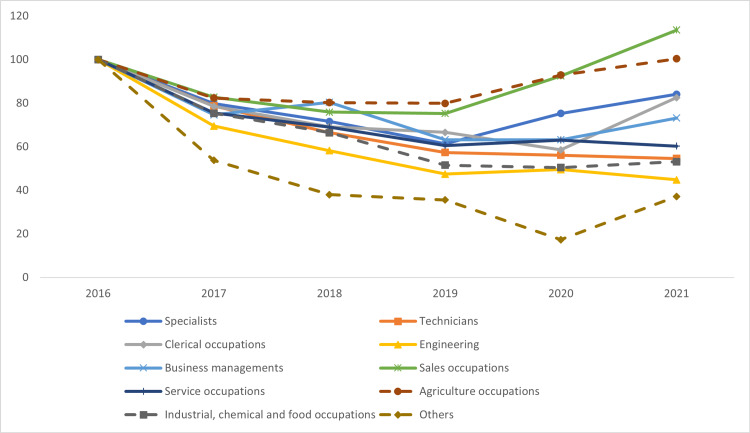
Trends in work-related injuries by occupation in Saudi Arabia between 2016 and 2021

In 2021, the highest percentage of injuries occurred among service occupations (47.5%), followed by engineering occupations (34.6%). However, the share of engineering occupation injuries decreased by 7.5% over the study period, while the share of service occupations increased by 3.6%. The number of injuries decreased across all occupations except the sales occupations, producing a slope (S) of 2.7, and in agricultural occupations, the injury number remained nearly steady, displaying only a slight increase (Figure 5).

In the injury-to-worker ratio, a decrease occurred in all occupations except in sales occupations, which displayed an increase with S = 5.4, almost identical to the increase in agriculture occupations (Table 5).

**Table 5:** Distribution of injury-to-worker ratio by occupation in Saudi Arabia between 2016 and 2021

Occupations	2016	2017	2018	2019	2020	2021
Specialists	0.00182	0.00147	0.00135	0.00120	0.00158	0.00124
Technicians	0.00510	0.00414	0.00377	0.00354	0.00381	0.00330
Clerical occupations	0.00083	0.00067	0.00058	0.00060	0.00081	0.00075
Engineering	0.00716	0.00543	0.00535	0.00541	0.00598	0.00494
Business managements	0.00099	0.00074	0.00078	0.00064	0.00080	0.00070
Sales occupations	0.00095	0.00078	0.00069	0.00072	0.00122	0.00106
Service occupations	0.00505	0.00385	0.00381	0.00388	0.00419	0.00375
Agriculture occupations	0.00269	0.00240	0.00273	0.00331	0.00427	0.00377
Industrial, chemical, and food occupations	0.00710	0.00565	0.00565	0.00523	0.00561	0.00561
Others	0.00047	0.00022	0.00019	0.00008	0.00002	0.00027

Distribution of work-related injuries by cause

**Figure 6:**
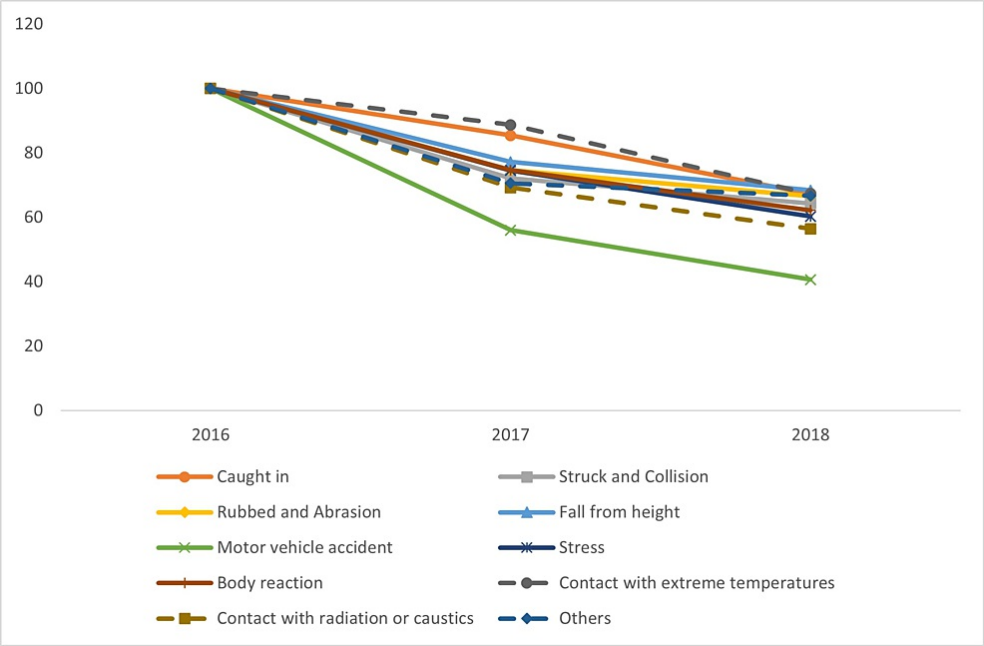
Trends in work-related injuries by injury cause in Saudi Arabia between 2016 and 2018

Regarding injury causes, this study covered two periods, 2016-2018 and 2020-2021. During the first period, falls were the most common causes, producing 28.5% of injuries in 2018, followed by struck and collision injuries (26.4%). In the second period, exposure to inanimate mechanical forces was the most common injury cause (46%) in 2021, followed by falls (37%). All cause indices decreased in the first period, with slope that ranges from -15.8 to -29.7, with the greatest decrease in motor vehicle accidents and the least decrease in falls. In the second period, there was a significant increase in transport accidents; exposure to electric current, radiation, and extreme ambient air temperature and pressure; and assault between 2020 and 2021. Generally, other causes decreased in 2021 (Figure 6 and Table 6).

**Table 6:** Distribution of work-related injuries by injury cause in Saudi Arabia (2020-2021)

Causes	2020 (%)	2021 (%)	Slope
Contact with heat and hot substances	704 (2.4)	645 (2.3)	-8.38
Assault	85 (0.3)	94 (0.3)	10.59
Overexertion, travel, and privation	93 (0.3)	69 (0.2)	-25.81
Accidental poisoning from and exposure to noxious substances	32 (0.1)	35 (0.1)	9.38
Exposure to electric current, radiation, and extreme ambient air temperature and pressure	246 (0.8)	308 (1.1)	25.20
Exposure to forces of nature	266 (0.9)	177 (0.6)	-33.46
Exposure to animate mechanical forces	1,083 (3.7)	1,022 (3.7)	-5.63
Exposure to inanimate mechanical forces	13,213 (45.8)	12,675 (46)	-4.07
Exposure to smoke, fire, and flames	342 (1.2)	346 (1.2)	1.17
Transport accidents	1,296 (4.5)	1,847 (6.7)	42.52
Falls	11,110 (38.6)	10,197 (37)	-8.22
Accidental drowning and submersion	2 (0.01)	5 (0.02)	150.00
Accidental threats to breathing	341 (1.2)	140 (0.5)	-58.94

Distribution of work-related injuries by injury status

**Figure 7:**
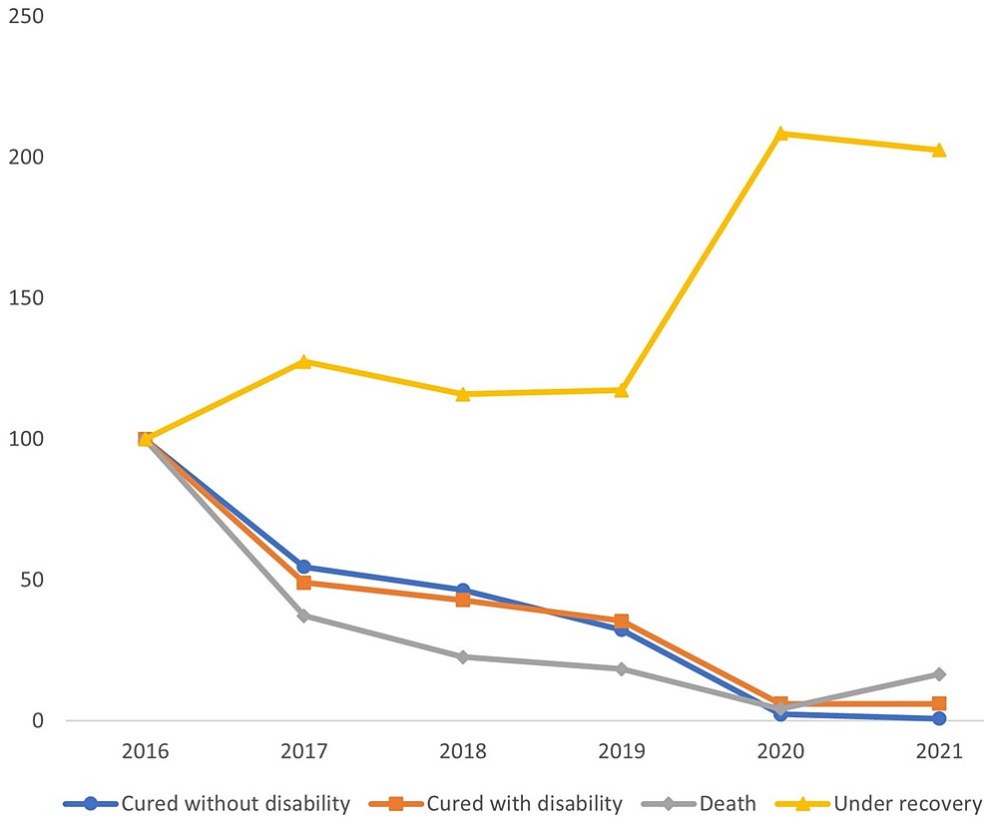
Trends in work-related injuries by injury status in Saudi Arabia between 2016 and 2021

The injury share of recovery without disability decreased from 66.4% in 2016 to only 1% in 2021, while the share of under-recovery status increased from 26.3% in 2016 to 98% in 2021. The indices of all statuses decreased significantly except the under-recovery status, which more than doubled (Figure 7).

## Discussion

This study revealed a decrease in work-related injuries in Saudi Arabia from 2016 to 2021. Additionally, the number of foreign workers decreased significantly during this period, indicating the effect of work policy changes in the country since 2016; one of these policies is the expatriate fee, which was implemented in 2017 [23]. The decrease in injuries is consistent with what Abbas et al. reported regarding the period from 2004 to 2016 [16]. It is worth noting that the COVID-19 pandemic forced public lockdowns in 2020, affecting many sectors and making 2020 the year with the lowest number of reported deaths.

Demographic distribution of work-related injuries

While injuries decreased across all age categories over the research period, the trend was less pronounced for workers older than 30; moreover, the proportion of injuries among workers older than 30 is rising. Evidence like this suggests that the new work policies and fees for foreign workers have reduced the opportunities available to those with less experience.

The research confirmed findings from previous research conducted in Saudi Arabia, Qatar, and the United Arab Emirates, which showed that foreign employees made up a much larger proportion of the labor force in those countries than did local nationals [13,24,25]. In other words, the injury-to-work ratio was significantly greater for non-Saudi workers than for Saudi workers, indicating that the former group performed more dangerous labor. In recent years, however, as the number of Saudi workers has grown, the disparity between the two categories has shrunk.

Regarding gender distribution, in the injury-to-worker ratio data, male workers were at far higher risk of work-related injuries than female workers. This fact held true even with the increase in female injuries in 2021, which was attributed to an increase in female workers. It is possible that this was caused by the conservative structure of Saudi society, which means that positions that require working outside in potentially dangerous conditions are virtually often held by males.

Distribution of work-related injuries by economic sector

There was a decrease in all sectors' injuries, except for agriculture and fishing, where increases occurred in 2020 and 2021. These changes may have occurred because the response to supply chain disruption during the COVID-19 pandemic necessitated an increase in agricultural activity to meet the national demand. Such an increase also appeared in the injury-to-worker ratio.

The construction industry continued to have the highest incidence of injuries, addressed in prior studies, but the proportion of construction injuries decreased significantly, from 51% in 2015, according to Mosly [17], to 45.1% in 2016 and 41.6% in 2021. Additionally, the injury-to-worker ratio decreased in the construction sector. This decrease was evident in the enforcement of safety at work sites and the collaboration of multi-government sectors to enhance the safety of construction sites. However, it is important to take into account what Abukhashabah et al. found to be effective in boosting workplace safety. This includes having a qualified safety and health supervisor on-site, maintaining equipment and machinery, using the right personal protective equipment (PPE), constantly training employees, and raising their level of awareness [26].

From 2020 to 2021, there was an increase in the community and social service sector injury shares, partially attributable to COVID-19's spread among healthcare workers.

Distribution of work-related injuries by occupation

Injury rates in the service sector were the highest during the research period, followed by those in engineering and technology. In contrary to what Abbas et al. found [16], engineers are not the most at-risk group for workplace accidents, with technicians coming in second. Since the injury-to-worker ratio fell for the service sector, the rise in injuries in this sector can be attributed to a rise in the number of service workers.

During the study period, the number of injuries in sales nearly doubled. This increase also appeared in the injury-to-worker ratio for this sector. This result warrants investigating the causes of such increases in future studies.

Distribution of work-related injuries by cause

The change of classification systems between the two periods examined made it difficult to trace trends throughout the study period. However, the fall category was the leading injury cause from 2016 to 2018 by shares (an increase from 26.8% to 28.5% of all injuries). This share increased significantly in the second period, 2020-2021, to 38.6% in 2020. However, the fall category was not the leading injury cause in the later period, instead falling below the exposure to inanimate mechanical force category. Since the new classification subdivided useful information into several categories, the absence of the other categories may have contributed to these shifts.

Distribution of work-related injuries by injury status

As indicated previously, death incidences reduced dramatically during the study period, as did the percentage of cured with disability status. However, the large growth in the under-recovery status, particularly during the years 2020-2021, may imply negligence in data collecting and data input on the part of the GOSI, and additional care is required when managing such sensitive data.

## Conclusions

This study revealed that work-related injuries decreased during the study period, continuing the trend addressed in similar studies. Additionally, there was a decrease in insured workers during the same period because of stricter government migration policies and taxes. However, noninsured workers were beyond the scope of this study since there were no clear specific data on them. Therefore, additional research should be undertaken on illegal worker injuries in Saudi Arabia, as these workers are more prone to work-related injuries than legally insured workers due to the absence of safe working settings.

This study demonstrated the growing numbers of Saudi workers in work markets and slightly higher injury rates among Saudis than before. However, the disparity between non-Saudi and Saudi workers is still too large because injury risks for non-Saudi workers are still significantly higher than for Saudi workers.

The main limitation of the study was that the GOSI database limitations should be addressed, especially regarding injury status and causes, since this information is valuable in determining work safety trends in the Kingdom of Saudi Arabia.
